# The Effect of Perinatal Exposure to Cafeteria Diet and Physical Activity on Diet Preference, Anxiety-like and Depressive-like Behavior, and Memory in Female and Male Offspring Rats

**DOI:** 10.3390/nu18132175

**Published:** 2026-07-04

**Authors:** Ana Karen Urbina-Rivera, María Elena Chávez-Hernández, Fernanda García-Rivas, Mariana Malpica-Gómez, Cecilia Ramírez-de-la-Vega, Sara Elisa Castañeda-Gómez, Luis Miguel Rodríguez-Serrano

**Affiliations:** Facultad de Psicologia, Universidad Anahuac Mexico, Huixquilucan 52786, Mexicoelena.chavez@anahuac.mx (M.E.C.-H.);

**Keywords:** cafeteria diet, physical activity, offspring rats, anxiety-like, depressive-like, memory

## Abstract

**Background/Objectives**: Overweight and obesity have consistently increased in prevalence. Early exposure to foods high in fats and sugar through maternal conditions may increase vulnerability to developing metabolic diseases and cognitive impairments in adulthood. In this regard, we aim to evaluate the effects that perinatal exposure to cafeteria diet (CAF) and physical activity (PA) has on anxiety-like, depressive-like behavior, memory and diet preference in male and female offspring. **Methods**: Seventy female and male offspring rats were divided into five groups according to maternal conditions: (1) CONTROL, fed only standard diet (SD) with no voluntary PA, (2) SED+SD, fed only SD with no voluntary PA, (3) SED+CAF, fed SD and CAF with no voluntary PA, (4) PA+SD, fed only SD with voluntary PA, and (5) PA+CAF, fed SD and CAF with voluntary PA. Starting on PND 24, offspring rats were exposed to SD and CAF (except for rats from the CON maternal group) and evaluated for seven weeks for diet preference, and at week seven for anxiety-like, depressive-like behavior and memory. **Results**: After seven weeks of exposure to CAF, maternal conditions showed significantly different effects on adult male and female offspring for diet preference and memory impairments. Furthermore, maternal PA significantly reduced anxiety-like and depressive-like behaviors in the offspring. **Conclusions**: Our results suggest that maternal conditions and postweaning CAF exposure have a joint influence on diet preference, anxiety-like and depressive-like behavior. Additionally, perinatal CAF exposure impairs memory in male and female offspring, regardless of maternal PA conditions. However, maternal PA was associated with reduced affective behaviors induced by lifelong CAF, presenting as a promising non-pharmacological intervention to promote favorable long-term behavioral outcomes in offspring.

## 1. Introduction

The World Health Organization (WHO) defines obesity as an abnormal or excessive accumulation of body fat that may pose a health risk. In recent decades there has been a concerning increase in the prevalence of overweight and obesity, with one of the main causes being diet composition. In particular, the excessive consumption of highly palatable, energy-dense foods high in sugar and fat (often consumed in the forms of “junk” or “fast” food) plays a central role in the development of obesity and its current pandemic status [1,2,3].

To study these effects, the cafeteria diet (CAF) animal model is designed to imitate high-calorie and low-nutrient ultraprocessed (UP) products that humans consume [4]. This model includes distinct orosensory properties (such as smell and texture) and high palatability to promote overconsumption, and is characterized by its dietary variety [4,5]. For instance, Fam et al. [6] demonstrated that CAF consumption in rats induces a stronger preference for high-sugar solutions, such as 32% sucrose, compared to low-sugar solutions, such as 2%; curiously, this preference decreases when compared to intermediate concentrations, such as 8%, reflecting alterations in sweetness sensitivity. Additionally, obesity-prone rats have been found to have lower sensitivity to mild sweet tastes alongside reduced expression of the T1R3 receptor, which may drive them to consume more calories when exposed to high-energy diets [7]. Also, CAF reliably induces obesity and promotes significant weight gain during developmental stages due to the consistent overconsumption of energy-rich foods [4,8,9]. Furthermore, CAF intake is associated with adverse metabolic outcomes, including insulin resistance, fat accumulation, and inflammation in the liver and adipose tissue, which are characteristics similar to the metabolic syndrome in humans [10].

Consuming CAF during early life stages, such as childhood and adolescence, produces more pronounced consequences than it does in adults [11,12]. Evidence indicates that hypercaloric diets during gestation and lactation can program the offspring brain, altering the structural and functional organization of reward circuits [13]. In this regard, animal studies show that early exposure to UP foods starting at weaning leads to accelerated weight gain, alterations in glucose metabolism, and changes in body composition [4,14]. Furthermore, early exposure to CAFs can exert lasting effects, establishing a pattern of vulnerability that persists into later life and increasing the likelihood of developing metabolic diseases and cognitive impairments in adulthood [14,15]. In this regard, an association has been found between CAF consumption and deficits in spatial memory and learning, and a higher predisposition to anxiety- and depressive-like behaviors [16,17,18], with neurobiological alterations that may be related to reduced motivation for natural rewards in the offspring [13,19]. Furthermore, a maternal high-fat diet has been reported to alter the dopamine and opioid systems of offspring at both weaning and adulthood [20].

Maternal physical activity (PA) during pregnancy and lactation has been proposed as a key factor in shaping offspring health [21]. Evidence from rodent studies suggests that when dams engage in regular exercise, they not only improve their own metabolic profile but also create a more favorable environment for their pups [22]. For instance, low-intensity maternal exercise has been shown to reduce adiposity, enhance glucose regulation, and modify milk composition, changes that may have long-lasting benefits by lowering the offspring’s susceptibility to metabolic dysfunction later in life [22]. Moreover, the influence of maternal exercise extends beyond metabolic outcomes to behavioral traits related to diet [23]. Research shows that rats born to exercising mothers display healthier body weight trajectories and a reduced preference for high-fat foods, indicating that maternal PA may mitigate the adverse effects of perinatal exposure to high-fat by adjusting reward pathways in the brain and influencing appetite regulation [20].

While studies indicate that maternal conditions play a critical role in offspring metabolic and behavioral development, the long-term effects of maternal exposure to a CAF with or without concurrent PA have not yet been fully explored. This presents as an important issue given the rising rates of overweight and obesity in children and adolescents, and also throughout adulthood. Furthermore, it is essential to evaluate if perinatal PA exposure acts as a protective factor for offspring when mothers are exposed to CAF during pregnancy and lactation. Additionally, understanding the role that maternal perinatal conditions play in the dietary preferences and behavioral phenotypes of both male and female offspring is highly relevant for evaluating long-term vulnerability. Therefore, the aim of the present study was to evaluate the effects of perinatal exposure to cafeteria diet and physical activity on anxiety-like and depressive-like behavior, memory and diet preference in male and female offspring. We hypothesized that perinatal exposure to CAF would impair memory and increase anxiety-like and depressive like behaviors, as well as increase preference for CAF. We also hypothesized that maternal PA would serve as a protective factor, mitigating the adverse effects of perinatal CAF exposure, as well as postweaning CAF intervention.

## 2. Materials and Methods

### 2.1. Subjects

Seventy male and female offspring (35 female and 35 male) from confirmed first-pregnancy female Sprague Dawley rats were divided into one of five groups (n = 7 per group) according to maternal perinatal conditions: (1) CON, fed only standard diet (SD) with no voluntary physical activity (PA), (2) SED+SD, fed only SD with no voluntary PA, (3) SED+CAF, fed SD and cafeteria diet (CAF) with no voluntary PA, (4) PA+SD, fed only SD with voluntary PA, and (5) PA+CAF, fed SD and CAF with voluntary PA. The PA intervention consisted of dams granted 24 h free access to an in-cage running wheel throughout the exposure period. Mother rats were kept on their assigned conditions during pregnancy and until PND 14 to avoid exposure of offspring to maternal conditions (CAF and/or PA). On PND 21 (weaning), 14 pups (seven female and seven male) per group were selected randomly from three breeding dams of the same perinatal conditions, to maximize cohort utility and minimize surplus animal generation, keeping the “Reduction” principle of the 3Rs bioethical standard [24]. This sample size was determined using G*Power software with the following parameters: effect size = 0.5; ∝ error probability = 0.05; power (1 − beta error probability) = 0.80; number of groups = 5; number of measurements = 7 (representing the seven weeks of experimental protocol). Animals were systematically stratified and housed in groups of 2–3 same-sex littermates per cage, ensuring a balanced representation from each maternal lineage. This approach allowed maintaining uniform housing and handling conditions, minimizing unnecessary stress in the experimental animals. Starting on PND 24, offspring were exposed to 7 weeks of both SD and CAF, except for offspring from the CON group who were kept strictly on a postweaning SD regimen, to evaluate preference after maternal conditions. All animals were housed in groups in a humidity- and temperature-controlled vivarium (22 °C ± 1 °C) with a 12:12 h light–dark cycle (lights on at 7:00 am [ZT0]; lights off at 7:00 pm [ZT12]).

### 2.2. Materials

#### 2.2.1. Cafeteria Diet

All animals had ad libitum access to water, SD (Nutricubos Purina^®^; 3.02 kcal/g; 23.0% protein, 3.0% fat, and 6.0% fiber) and CAF. CAF consisted of a combination of sweet and salty UP foods listed in Table 1: hot dog sausages (FUD ^®^; 10.2% fat, 6.6% carbohydrates, 10.6% protein), Oreo^®^ cookies (Nabisco^®^; 19.7% fat, 68.4% carbohydrates, 5% protein), potato chips (Great Value^®^; 26.6% fat, 51.7% carbohydrates, 8.1% protein), KitKat^®^ chocolate (Nestlé ^®^; 29.86% fat, 57.36% carbohydrates, 7.9% protein), salty crackers (Great Value^®^; 23.7% fat, 59.6% carbohydrates, 6.8% protein), and mini chocolate marshmallows (Great Value ^®^; 14.5% fat, 72.8% carbohydrates, 4.2% protein). On average, the food items contained 17.48% of fat, representing a high-fat, energy-dense composition.

#### 2.2.2. Elevated Plus Maze

The elevated plus maze test was used to measure anxiety-like behavior. The apparatus consists of a plus-shaped maze elevated above the ground, with four arms extending from a central platform: two are open, while the other two are enclosed by walls. The animal is placed in the central square and allowed to freely explore for five minutes. Time in open arms and time in closed arms were analyzed [26].

#### 2.2.3. Open Field Test

The open field test (OFT) was used to evaluate anxiety and locomotion. Animals were placed in a 50 × 50 × 50 cm squared arena and allowed to freely explore for 5 min. Anxiety and locomotor activity were analyzed using total distance traveled and proportion of time spent in the center zone as parameters. Furthermore, this task was used to habituate the mice for the Novel Object Recognition Task (NORT) [26,27].

#### 2.2.4. Novel Object Recognition

Memory was evaluated using the novel object recognition test (NORT) in both short- and long-term memory. The task uses a 50 × 50 × 50 cm squared arena where the animal is free to explore for 5 min two objects for three phases: (1) acquisition (ACQ), in which two identical objects are placed in the center of the arena, equidistant from each other and from the walls; (2) short-term memory (STM, 30 min after the ACQ phase), where one of the objects is replaced with a new one different in shape, color and texture; and (3) long-term memory (LTM, 24 h after the ACQ phase, where the object used for the STM phase is replaced with another one different in shape, color and texture. Discrimination ratio (DR) for each familiar (FAM) and novel (NOV) object was computed as follows:

DR = (Time exploring object/total exploration time)

where total exploration time refers to the sum of time exploring NOV and FAM objects.

#### 2.2.5. Marble Burying Test

The marble burying test (MBT) is a behavioral assay used to evaluate responses associated with anxiety. The test involves placing the animal in a 20 × 30 cm acrylic cage with approximately 5 cm of clean wood chip bedding and arranging twenty 15 mm diameter glass marbles in five lines of four marbles each, equidistant from each other and from the cage walls. The animal is allowed to interact freely for 30 min. The number of marbles buried up to two-thirds is quantified [28].

#### 2.2.6. Forced Swim Test

The forced swim test (FST) was used to measure depression-like behavior. Animals are placed in a clear glass cylinder for six minutes filled with water at a 24 ± 1 °C. Total time immobile in seconds was used as a parameter [29,30].

### 2.3. Procedure

**Figure 1:**
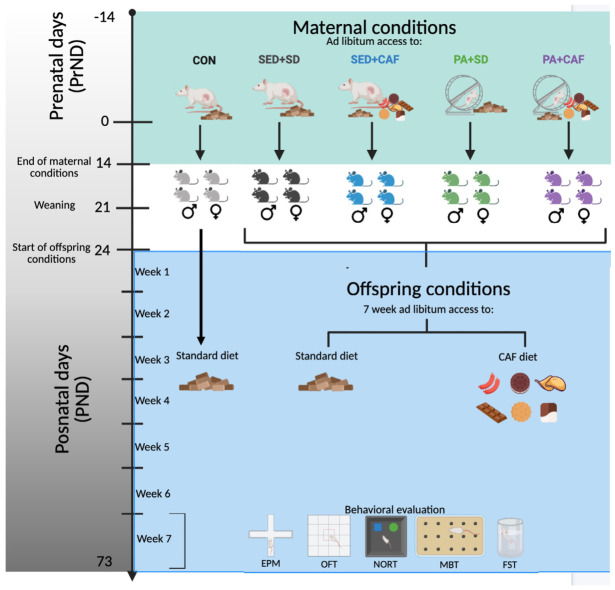
Experimental timeline. Confirmed first-pregnancy female Sprague Dawley rats were divided into one of five groups (n = 7 per group for each sex): (1) CON, (2) SED+SD (3) SED+CAF (4) PA+SD, and (5) PA+CAF. Starting on PND 24, offspring were exposed for seven weeks to ad libitum access to SD and CAF diet. After seven weeks, animals were evaluated in behavioral tasks. PrND: prenatal day; PND: postnatal day; SD: standard diet; CAF: cafeteria diet; EPM: elevated plus maze; OFT: open field test; NORT: novel object recognition test; MBT: marble burying test; FST: forced swim test. Figure made with BioRender.com.

Figure 1 shows the experimental timeline. Confirmed first-pregnancy female Sprague Dawley rats were randomly allocated into one of five groups based on the perinatal conditions to which they were exposed from prenatal day (PrND) 14 to postnatal day (PND) 14: (1) CON, fed only standard diet (SD) with no voluntary physical activity (PA), (2) SED+SD, fed only SD with no voluntary PA, (3) SED+CAF, fed SD and CAF with no voluntary PA, (4) PA+SD, fed only SD with voluntary PA, and (5) PA+CAF, fed SD and CAF with voluntary PA. Rats were kept on their assigned conditions during pregnancy and until PND 14 to avoid exposure of offspring to maternal conditions (CAF and/or PA). On PND 21 (weaning), 14 pups (seven female and seven male) per group were selected randomly from three breeding dams of the same perinatal conditions, to maximize cohort utility and minimize surplus animal generation. Animals were systematically stratified and housed in groups of 2–3 same-sex littermates per cage. Starting on PND 24, offspring were divided into the same groups of their maternal conditions per sex (female [FE] and male [MA]) and were exposed for seven weeks to both SD and CAF, except for offspring from the CON group who were maintained strictly on a postweaning SD, to evaluate diet preference after maternal conditions. Offspring from the CON group (maintained strictly on lifelong SD) were included to compare with offspring rats with lifelong exposure to SD and CAF in behavioral tasks, to differentiate if the effects found were due to maternal conditions and/or lifelong CAF exposure.

To analyze the preference index (PI) for total CAF, salty CAF and sweet CAF, daily diet PI was calculated for each element. First, intake in grams was obtained for SD and CAF by subtracting from the weight of each food placed (WFplaced) the weight of the food found (WFfound) 24 h later (WFplaced–WFfound). Then, total kilocaloric intake (Totalkcal) was obtained by multiplying grams consumed for each component (SD or CAF) by the kilocalories per gram for each diet element (see Table 1), and then the kilocalories of grams consumed for CAF (CAFkcal) and standard diet (SDkcal) were added. Finally, daily PI for total CAF (PI CAFtotal), salty CAF (PI CAFsalty), and sweet CAF (PI CAFsweet) was calculated as follows:

PI CAFtotal = TotalCAFkcal/(Total kcal)

PI CAFsalty = SaltyCAFkcal/(Total kcal)

PI CAFsweet = SweetCAFkcal/(Total kcal)

In week seven, behavioral testing was performed to evaluate anxiety-like, depressive-like behaviors, and memory in the following sequential order: (1) EPM, (2) OFT, (3) NORT, (4) MBT, and (5) FST. All behavioral tasks were performed during the light phase between 10:00 am and 3:00 pm (depending on the number of animals tested). Furthermore, the apparatus was thoroughly cleaned with a 70% ethanol solution between trials to eliminate olfactory cues. Automated video tracking and analysis from EPM, OFT, NORT and FST were performed using ANY-maze^®^ Software version 7.65 (Stoelting Europe Co^®;^ Wood Dale, IL, USA) to eliminate observer bias by an experimenter blinded to the treatment groups. For the MBT, the number of marbles buried up to two thirds of their circumference was manually quantified by experimenters blinded to the experimental conditions.

### 2.4. Statistical Analysis

Data were prepared in Excel and are reported as the mean ± standard error of the mean (SEM). Results were analyzed using Graph Pad Prism (San Diego, CA, USA) version 9.3.1 (350) (GraphPad Software LLC, 2021). Figures were generated using GraphPad Prism^®^. Primary outcomes analyzed were PI for sweet, salty and total CAF, and behavioral results from EPM, OFT, MBT, FST and NORT.

The PI for CAF is represented as the mean daily proportion of kcal CAF intake per day, per week. PI was computed per week for total CAF, salty CAF, and sweet CAF. Two-way repeated measures ANOVA (group × week) with Sidak’s multiple comparisons test (∝ < 0.05) was used to evaluate the main effect of maternal group (group) and time, as well as their interaction effect.

To evaluate results from EPM, OFT, FST, MBT behavioral tasks, one-way ANOVA with Sidak’s multiple comparisons test (∝ < 0.05) was performed. Additionally, NORT results were analyzed with a two-way mixed effects ANOVA (group × object) with Sidak’s multiple comparisons test (∝ < 0.05) to compare the main effects of maternal group and DR in exploration time for each object in STM and LTM stages.

For all the main one-way and two-way ANOVA results, effect sizes are represented as “% of variance explained” which corresponds to the Eta-squared value calculated by the statistical software. All statistical analysis for CAF PI and behavioral tasks results were performed separately for female and male offspring. The CON group was included in behavioral task analyses as a baseline reference standard and omitted from the PI for CAF analysis given that they were not exposed to this diet intervention.

### 2.5. Ethics Statement

All experiments were conducted in accordance the Mexican Official Norm NOM-062-ZOO-1999 technical specifications for the production, care, and use of laboratory animals, as well as with the National Institutes of Health guide for the care and use of laboratory animals (NIH Publications N°. 8023, revised in 1978) and local Mexican laws to minimize the number of animals used and their suffering. The present project was approved by the Dirección de Investigación, Universidad Anáhuac México with the ID number PI0000154.

## 3. Results

Results are presented for diet preference and each behavioral parameter analyzed for female and male offspring. Table 2 shows a summary of the main findings of the present study. Also, Supplementary Tables S1–S3 show detailed results for Sidak’s multiple comparisons tests including mean difference, 95% confidence intervals (CI) of difference, and adjusted *p* values.

### 3.1. Preference for Cafeteria Diet

**Figure 2:**
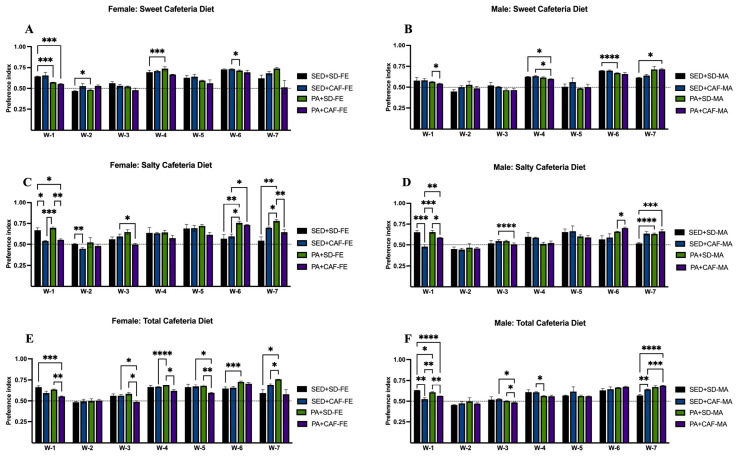
**Female and male offspring cafeteria diet preference index.** Data express the mean ± SEM. (**A**,**B**): Sweet cafeteria diet preference for female (**A**) and male (**B**) offspring. (**C**,**D**): Salty cafeteria diet preference for female (**C**) and male (**D**) offspring; (**E**,**F**). Total cafeteria diet preference for female (**E**) and male (**F**) offspring. Two-way repeated measures ANOVA (group × week) with Sidak’s multiple comparisons test (* *p* < 0.05; ** *p* < 0.01; *** *p* < 0.001; **** *p* < 0.0001). n = 7 per group for each sex.

Figure 2 shows the results of the female and male offspring preference index for sweet CAF (Figure 2A,B), salty CAF (Figure 2C,D) and total CAF (Figure 2E,F). For **sweet CAF PI** analysis, two-way repeated measures ANOVA (group × week) female offspring results indicate significant effects of time (F (1.377, 8.262) = 37.63; *p* = 0.0001; 49.75% variance explained) and group (F (1.596, 9.575) = 11.66; *p* = 0.0037; 5.714% variance explained), while no significant effect of interaction was found (“F (1.565, 9.392) = 2.637”; *p* = 0.1298; 9.868% variance explained). For male offspring, no significant effect of group (F (1.862, 11.17) = 1.146; *p* = 0.3486; 0.7084% variance explained) or interaction (F (1.943, 11.66) = 1.839; *p* = 0.2026; 6.741% variance explained) was found, but there was a significant effect of time (F (1.572, 9.435) = 63.91; *p* < 0.0001; 59.68% variance explained). Sidak’s multiple comparisons test results for female offspring indicate significant differences between groups in weeks 1, 2, 4 and 6 (Figure 2A; all *p* values < 0.05), while male offspring results indicate significant differences in weeks 1, 4, 6 and 7 (Figure 2B; all *p* values < 0.05).

For both female and male offspring, two-way repeated measures ANOVA (group × week) analysis results on **salty CAF PI** indicate significant effects of **interaction group × week (female: F (1.847, 11.08) = 6.818; *p* = 0.0129; 16.35% variance explained; male: F (2.001, 12.00) = 5.281, *p* = 0.0226, 18.44% variance explained)**, and time **(female: F (1.502, 9.012) = 27.12, *p* = 0.0003, 29.46% variance explained; male: F (1.828, 10.97) = 18.44, *p* = 0.0004, 34.10% variance explained)**, while there was no significant effect of group **(female: F (1.175, 7.047) = 4.235, *p* = 0.0748, 11.39% variance explained; male: F (1.726, 10.36) = 0.3372, *p* = 0.691, 0.5722% variance explained)**. Sidak’s multiple comparisons test indicates significant differences between groups in female offspring in weeks 1–3 and 6–7 (Figure 2C; all *p* values < 0.05), while for male offspring differences among groups are shown in weeks 1, 3 and 7 (Figure 2D; all *p* values < 0.05).

Regarding **total CAF** preference, female offspring two-way repeated measures ANOVA (group × week) analysis results indicate significant effects of time (F (1.464, 8.782) = 53.02; *p* < 0.0001; 49.00% variance explained) and interaction (F (2.159, 12.95) = 5.274; *p* = 0.0195; 11.09% variance explained), while no significant effect of group was found (F (1.112, 6.670) = 5.342; *p* = 0.0536; 8.656% variance explained). For male offspring, no significant effect of group (F (1.400, 8.402) = 0.2428; *p* = 0.713; 0.3158% variance explained) or interaction (F (1.811, 10.87) = 3.825; *p* = 0.0585; 11.43% variance explained) was found, but there was a significant effect of time (F (1.895, 11.37) = 44.29; *p* < 0.0001; 54.47% variance explained). Sidak’s multiple comparisons test results for female offspring indicate significant differences between groups in weeks 1 and 3–7 (Figure 2E; all *p* values < 0.05), while male offspring results indicate significant differences weeks 1, 3, 4 and 7 (Figure 2F; all *p* values < 0.05).

### 3.2. Behavioral Analysis Results

#### 3.2.1. Elevated Plus Maze

**Figure 3:**
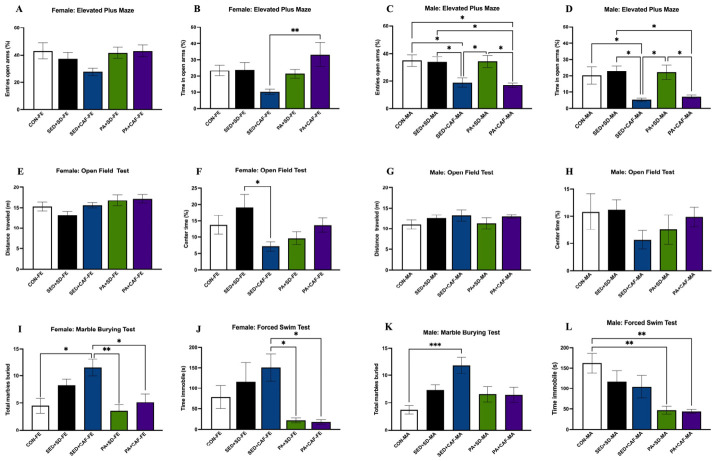
**Female and male offspring behavioral analysis results**. Data express the mean ± SEM. (**A**–**D**): Elevated plus maze results: proportion of entries (**A**,**C**) and proportion of time spent in open arms (**B**,**D**) for female and male offspring. (**E**–**H**): Open field results for male and female offspring: total distance traveled (**E**,**G**) and proportion of time spent in center zone (**F**,**H**). (**I**,**K**): Marble burying test results for female (**I**) and male (**K**) offspring. (**J**,**L**): Forced swim test results: total immobile time for female (**J**) and male (**L**) offspring. One-way ANOVA with Sidak’s multiple comparison analysis (* *p* < 0.05; ** *p* < 0.01; *** *p* < 0.001). n = 7 per group for each sex.

The elevated plus maze (EPM) was used to evaluate differences in anxiety-like behavior. Percentage of entries to open arms (Figure 3A for females and Figure 3C for males) and proportion of time spent in open arms (Figure 3B for females and Figure 3D for males) were analyzed as parameters. Male and female offspring were analyzed separately using one-way ANOVA analysis. The results of **female offspring** indicate no significant differences between maternal conditions groups in percentage of entries to **open arms (F (4, 29) = 2.179; *p* = 0.0963),** while there were significant differences in proportion of **time spent in open arms (F (4, 29) = 3.643; *p* = 0.016).** Sidak’s multiple comparisons test indicates that the SED+CAF+FE group spent significantly less time in open arms than the PA+CAF+FE group **(*p* = 0.006),** which could indicate that maternal exposure to PA may act as a protective factor for the effects of CAF on anxiety. Even though no additional significance was found, the results show that the SED+CAF+FE group tends to spend less time in open arms than the rest of the groups.

Regarding **male offspring** results from the EPM, one-way ANOVA analysis results indicate significant differences between groups in proportion of entries to **open arms (F (4, 30) = 6.394; *p* = 0.0008)** and time spent in open arms **(F (4, 30) = 6.074; *p* = 0.0011)**. Sidak’s multiple analysis results show that male offspring from the SED+CAF+MA and PA+CAF+MA groups have fewer entries to open arms and spend less time in open arms **(all *p* values < 0.05)**.

#### 3.2.2. Open Field Test

The open field test was used to evaluate locomotion and anxiety-like behavior. Total distance traveled was analyzed as a locomotion parameter (Figure 3E for females and Figure 3G for males). One-way ANOVA analysis results indicate that **female offspring** show no significant differences between groups of maternal condition in **total distance travelled** in **female (F (4, 29) = 2.402; *p* = 0.0727) and male (F (4, 30) = 0.8343; *p* = 0.5141)** offspring. To evaluate anxiety-like behavior, the proportion of time spent in the center was analyzed (Figure 3F for females and Figure 3H for males). One-way ANOVA analysis results show significant differences between groups for female offspring **(F (4, 29) = 3.068; *p* = 0.0318);** Sidak’s multiple comparison analysis indicates that the SED+CAF+FE group spent significantly less time in the center than the SED+SD+FE group **(*p* = 0.0228).** For male offspring, one-way ANOVA analysis showed no significant differences between groups in proportion of time spent in the center **(F (4, 30) = 1.005; *p* = 0.4206)**.

#### 3.2.3. Marble Burying Test

The marble burying test was used to evaluate anxiety-like behavior (Figure 3I for females and Figure 3K for males). The number of marbles buried up to two-thirds of circumference was used as a parameter. One-way ANOVA analysis results revealed significant differences between groups for both **female (F (4, 29) = 5.953; *p* = 0.0013) and male offspring (F (4, 30) = 5.392; *p* = 0.0022)**; Sidak’s multiple comparisons test in female offspring indicates that the SED+CAF+FE group buried significantly more marbles than the CON+FE, PA+SD+FE and PA+CAF+FE groups, while in male offspring the SED+CAF+MA group buried more marbles than the CON+MA group (all female and male *p* values ≤ 0.01).

#### 3.2.4. Forced Swim Test

To evaluate depressive-like behavior, immobile time (s) in the forced swim test (FST) was analyzed as a parameter (Figure 3J for females and Figure 3L for males). For both female and male offspring, one-way ANOVA analysis indicated significant differences between groups (female **F (4, 29) = 4.092; *p* = 0.0095; male F (4, 30) = 5.727; *p* = 0.0015).** Sidak’s multiple comparisons test showed significantly less time immobile in the PA+SD+FE and PA+CAF+FE groups compared to the SED+CAF+FE group (both *p* values < 0.05); for male offspring, the PA+SD+MA and PA+CAF+MA groups showed significantly less immobile time compared to the CON+MA group (both *p* values < 0.01). These results found in male and female offspring indicate that maternal PA may have a protective effect in depressive-like behavior.

#### 3.2.5. Novel Object Recognition Task

**Figure 4:**
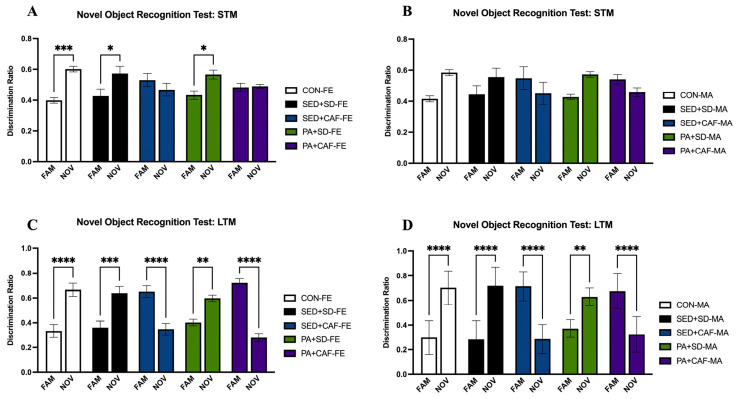
Novel object recognition test results. Data express the mean ± SEM. (A-B): Short-time memory results for female (**A**) and male (**B**) offspring. (**C**,**D**): Long-time memory results for female (**C**) and male (**D**) offspring. Two-way ANOVA analysis (group × time) with Sidak’s multiple comparisons test (* *p* < 0.05; ** *p* < 0.01; *** *p* < 0.001; **** *p* < 0.0001). n = 7 per group for each sex.

To evaluate memory, the short-term and long-term memory stages of the novel object recognition task (NORT) were used. Within each group, DR in exploration time for each familiar (FAM) and novel (NOV) object was analyzed as a parameter. NORT results for female and male offspring are shown in Figure 4.

For **short-term memory (STM)**, two-way ANOVA analysis results for **female offspring** (Figure 4A) indicate a significant effect of **FAM or NOV object (F (1, 58) = 15.80; *p* = 0.00002;** 16.93% variance explained) and interaction **(F (4, 58) = 5.132; *p* = 0.0013;** 21.99% variance explained), while there was no significant effect of group (**F (4, 58) = 0.05452; *p* = 0.9943;** 0.2336% variance explained). Sidak’s multiple comparison analysis results indicate that for female offspring, the CON+FE, SED+SD+FE and PA+SD+FE maternal condition groups show significantly higher DR for the NOV object (all *p* values < 0.05). For **male offspring**, two-way ANOVA analysis results on **STM stage** (Figure 4B) indicate no significant effect of **group (F (4, 58) = 1.931 × 10^−31^; *p* > 0.9999;** 1.013 × 10^−30^% variance explained) or **FAM or NOV object (F (1, 58) = 2.786; *p* = 0.1005;** 3.656% variance explained)**,** while there was a significant effect of interaction (**F (4, 58) = 3.950; *p* = 0.0067;** 20.73% variance explained). However, Sidak’s multiple comparison analysis results indicate no significant differences in DR for NOV and FAM objects when adjusting *p* values.

Regarding **long-term memory (LTM) results**, two-way ANOVA analysis for **female offspring** (Figure 4C) indicates no significant effect of group (**F (4, 58) = 4.408 × 10^−3^^1^; *p* > 0.9999;** 9.024 × 10^−31^% variance explained) **or FAM or NOV object (F (1, 58) = 0.2106; *p* = 0.6480;** 0.1078% variance explained), but a significant effect of interaction **(F (4, 58) = 5.132; *p* = 0.0013;** 21.99% variance explained)**. For male offspring (Figure 4D), there was a significant effect of FAM or NOV object (F (1, 60) = 4.315; *p* = 0.0421;** 2.004% variance explained) and interaction **(F (4, 60) = 37.74; *p* < 0.0001;** 70.13% variance explained), and no significant effect of group (**F (4, 60) = 0.000; *p* > 0.9999;** 0.00% variance explained). Sidak’s multiple comparison analysis results indicate that both female and male offspring from the CON, SED+SD and PA+SD maternal conditions groups have significantly higher DR for the NOV object (all *p* values ≤ 0.01), while the SED+CAF and PA+CAF male and female groups have significantly lower DR for the NOV object (all *p* values ≤ 0.01) in this memory stage.

## 4. Discussion

The present study aimed to evaluate the effect of perinatal exposure to CAF and PA in anxiety-like, depressive-like behavior, memory and diet preference in male and female offspring. Our results show that both CAF and maternal PA exert distinct influences on the emotional-like conducts and cognitive factors of the offspring, with significant differences in both sexes. Overall, the results are consistent with previous research that indicates that CAF and hyperpalatable food negatively affect emotional regulation, increase vulnerability to stress, and impair cognitive processes related to memory [4,10,18].

### 4.1. Maternal Conditions Have Sex-Specific Effects in Anxiety-like and Depressive-like Behavior

Female offspring generally displayed lower anxiety-like behaviors compared to the male offspring, a consistent pattern with reports showing greater male vulnerability to anxiety and stress alterations induced by CAFs [4,31]. Additionally, results indicate that male offspring from CAF maternal conditions groups, regardless of their activity condition, showed the highest anxiety levels during the EPM task, significantly when compared with SD groups (PA+SD and SED+SD), which is consistent with previous studies that show how anxiety is elevated in male rats when they are exposed to CAF in early stages [32]. Furthermore, PA+CAF offspring spent a significant longer time in open arms during the EPM task in comparison to the SED+CAF group, indicating that PA is related to improved offspring’s metabolic regulation and modulates behavioral outcomes [22,33].

On the other hand, results from the OFT indicate that the female offspring from the group SED+CAF exhibited significantly higher levels of anxiety-like behavior; likewise, this tendency remained in the male offspring; these results are expected as CAF has been proved to elevate anxiety-like behavior [27].

MBT was also conducted, and consistently with previous results, groups with PA conditions presented lower levels of anxiety-like behavior in both male and female offspring, being significantly lower compared with SED+CAF in females, a group that showed significantly higher results than the CON group, exhibiting how CAF and SED conditions affect anxiety in offspring [22,33].

The present study also shows the effects that CAF and PA have on depressive-like behaviors in offspring. In this regard, it is important to note that the FST indicates that both male and female offspring from the groups exposed to PA (PA+SD and PA+CAF) have lower immobility time values compared to the SED groups (SED+SD and SED+CAF). This result is consistent with reports that state that maternal exercise reduces depression-like behavior in offspring regardless of their dietary condition [22,33]. It is important to mention that the recent literature proposes that immobility in the FST could be an indicator of a shift from active to passive coping strategies when faced with an important stressor [34]. In this regard, our results suggest that maternal PA may exert a protective effect that promotes resilient, active coping behavior, mitigating vulnerability towards passive coping behaviors induced by lifelong CAF exposure.

Alterations in PA, reduced social behaviors, and the emergence of anxiety- and depressive-like symptoms have been observed, with males showing greater vulnerability than females [4,31]. In this regard, Lippert et al. [35] have shown that the modulation of the dopaminergic pathway by maternal diet can be expressed differently depending on sex, with females exhibiting more pronounced changes in hedonic response.

### 4.2. Memory Impairment Through Lifelong Exposure to CAF

Early-life exposure to CAF has been reported to induce impairments in memory, as shown in the NORT results [17,18]. Both male and female offspring from the groups exposed to CAF show impaired memory, indicating that said diet exposure is a strong risk capable of disrupting cognitive processes even in the presence of protective maternal PA. These results are consistent with evidence that high-fat and high-sugar diets alter synaptic plasticity and learning-related pathways [36]. Furthermore, it is important to note that memory is significantly preserved in male and female SD maternal conditions, indicating that perinatal diets may mitigate adverse effects in memory as a result of lifelong exposure to CAF [37,38]. Additionally, it has been shown that maternal CAF consumption induces long-term memory impairment by imbalance brain-derived neurotrophic factor (BDNF) [39], and MAPK and PI3K in offspring [40]. Specifically, evidence suggests that BDNF has a role in regulating energy homeostasis in response to feeding and PA [41]. In this regard, our results suggest that maternal PA without CAF exposure may modulate the effects of CAF in offspring, leading to better performance in memory tasks when compared to offspring whose mothers were SED and/or were exposed to CAF.

### 4.3. Maternal Conditions Influence Preference for Cafeteria Diet

Regarding total CAF preference across weeks, significant effects of maternal diet and PA were observed in both male and female offspring. Similar patterns were also observed when sweet and salty CAF preferences were analyzed separately. In females, significant differences between groups were present during weeks 1, 3, 4, 5, 6, and 7, suggesting that females may be more susceptible to the reinforcing effects of diets high in palatable foods, largely due to the influence of reproductive hormones on reward circuits [42]. This could explain why females show greater variability between experimental conditions. In addition to the above, it has been reported that females react in a more limited way when stress, lack of PA, and palatable foods are combined, resulting in greater preference or consumption [43].

Also, offspring from the PA+CAF group presented a significantly lower PI for CAF when compared to the SED+CAF group, a tendency that remained consistent when analyzing sweet and salty CAF preference independently. This is consistent with previous reports that state that exposure to high-fat diets during lactation generate functional and structural changes in the development of regulative centers of the appetite in the hypothalamus [44]. Furthermore, exercise has been reported to decrease motivation toward highly reinforcing stimuli, as it can transform the reward system and thus decrease the search for palatable foods [45,46].

However, our results suggest that the exposure to CAF may mitigate the protective effects maternal PA can have during the perinatal phase. In addition, studies have proved that PA can have anxiolytic effects that increase the ingest of offspring, explaining the higher preference for CAF in the PA+SD group in females and in PA+SD and PA+CAF in males, patterns that were similarly reflected in both sweet and salty CAF preference [20,47]. Furthermore, male offspring results until week 3 show that groups from PA maternal conditions have a lower preference for CAF. Nonetheless, in weeks 6 and 7 this preference changed, and results indicate a significantly higher preference when compared specifically with the SED+SD group. This result was also identified in sweet and salty CAF preference, suggesting that the appearance of this preference may be possibly influenced by male endocrine maturation and lower stability in hedonic regulation, as well as a cumulative effect of the exposure to CAF. In this regard, Naef et al. [48] have shown that perinatal exposure to high-fat diets can generate dopaminergic modifications that fluctuate throughout development, with periods of normalization and reemergence in the response to reinforcing stimuli. Additionally, it has been shown that CAF also induced obesity and metabolic disruption of offspring, which led to alterations in hypothalamic functions such as hypothalamic–pituitary–gonadal [49]. Furthermore, maternal consumption of CAF has a structural effect in offspring rat brains, such as reduced volume in nucleus accumbens, hippocampus and prefrontal cortex, correlating to depression-like behavior [13], as well as reduced volume in the amygdala, increasing anxiety-like behavior [50].

It is important to note that every group, regardless of the maternal conditions they were exposed to, had a CAF preference higher than 50%, except in the second week of the experiment. This PI was observed not only in total CAF preference but also in both sweet and salty CAF preference, indicating that long-term exposure to CAF has risk effects in the dietary preference of offspring that cannot be eradicated by maternal conditions, especially when they start at early life stages. This result is consistent with previous studies that show the same patterns in preference regardless of the conditions the mothers had been exposed to [51]. Furthermore, it has been shown that maternal nutrition is a key factor influencing the postnatal health and disease susceptibility of offspring [52]. In this regard, nutritional and environmental factors, such as PA during the prenatal period, induce programming that modifies the structure and function of behaviors. In this regard, it has been identified that developmental plasticity by maternal nutritional conditions (specifically macro- and micronutrients) can induce changes and adapt to the natural postnatal environment while the offspring is still a fetus, optimizing its chances of survival into adulthood [52,53]. Additionally, maternal consumption of a high-fat diet promotes metabolic alterations in the offspring, while maternal exercise reduces these metabolic alterations [54]. Also, maternal nutrition (high-fat or junk foods) during pregnancy can alter the development of the reward system (opioid and dopamine systems) and program an increased preference for junk foods in the offspring [55,56]. Therefore, our results are consistent with the idea that maternal nutrition and PA induce developmental programming that modifies both behavioral structure and function.

## 5. Conclusions

The present results show that CAF alters both the preference for and motivation to consume high-fat and high-sugar foods, such as those included in a CAF model, contributing to the development and maintenance of obesity in these animal models. Our results are consistent with previous reports of vulnerability to the development of obesity via long-term exposure to hypercaloric foods, especially when exposure starts at early life stages, regardless of potentially protective factors in the perinatal period. Additionally, our study demonstrates that protective perinatal conditions lose impact throughout lifelong exposure to CAF. In this regard, maternal PA presents as a highly relevant potential protective factor in the perinatal period, particularly to anxiety- and depressive-like behaviors. However, even with this condition, exposure to CAF in the perinatal period, as well as throughout life, has a deteriorating effect on offspring memory evaluated in adulthood. This indicates that while maternal PA may protect against the affective outcomes considered in this study, it does not prevent long-term memory impairments associated with lifelong CAF exposure.

Some limitations in the present study should be acknowledged. First, assessing metabolic markers such as blood glucose and insulin levels in both lactating (PND 14–15) and weaned pups (PND 21) could provide an interesting insight into how perinatal maternal conditions program early-life metabolic health prior to CAF exposure. Furthermore, analyzing metabolic variables (blood glucose and insulin levels) alongside adipose tissue morphology in adult offspring following the seven weeks of postnatal CAF exposure would significantly complement our behavioral results. Also, while in the present study we characterized the behavioral outcomes of maternal and offspring interventions, correlating these dietary preferences and affective behavior results with specific molecular changes, such as dopamine receptor expression within the homeostatic and hedonic feeding brain areas (e.g., hypothalamus, nucleus accumbens and ventral tegmental area), remains an important variable to consider in future research. Finally, it is important to note that the statistical analysis in the present study considered individual offspring as the primary units of observation, rather than considering a potential confounding litter effect by employing the litter average method. While using individual rats as data points preserves the full range of behavioral and individual variance within cohorts, it introduces the influence of shared maternal environment (litter effect) as a potential confounding variable. In this regard, the housing of same-sex littermates together in cages sought to avoid unnecessary stress in the experimental animals. Furthermore, to mitigate potential environmental confounding variables, all housing conditions, rack placements and handling protocols were kept strictly uniform across all groups.

Taken together, evidence from the present study highlights the importance of maternal perinatal conditions, particularly the potential protective effect of PA, with or without CAF, as a promising non-pharmacological intervention to promote better long-term health outcomes in offspring in affective and behavioral outcomes. Furthermore, our findings highlight the importance of perinatal nutrition in shaping offspring diet preference from juvenile to adulthood periods, as well as its potential role in long-term susceptibility to overweight and obesity development.

## Figures and Tables

**Table 1:** List of cafeteria diet food items.

Order	Brand	Product	Kcal/g	Fat (%)	Protein (%)	Carbohydrates (%)
1	Fud ^®^	Hot dog sausages	1.61	10.2%	10.6%	6.6%
2	Nabisco ^®^	Oreo ^®^ cookies	4.709	19.7%	5.0%	68.4%
3	Great Value ^®^	Potato chips	4.786	26.6%	8.1%	51.7%
4	Nestle ^®^	KitKat ^®^ chocolate	5.30	29.86%	7.9%	57.36%
5	Great Value ^®^	Salty crackers	4.7885	23.7%	6.8%	59.6%
6	Great Value ^®^	Chocolate-covered mini marshmallows	4.385	14.5%	4.2%	72.8%

*Note*: food item macronutrient contents (fat, protein and carbohydrates) are reported as percentage by weight (g/100 g of product), in accordance with Mexican food labeling standards (NOM-051-SCFI/SSA1-2010 [25]).

**Table 2:** Summary of main findings of the study.

Variable	Behavioral Task	Parameter	Female	Male
**Diet preference**	**-**	Sweet	Y	Y
		Salty	Y	Y
		Total	Y	Y
**Anxiety-like behavior**	**EPM**	% entries to open arms	N	Y
		% time in open arms	Y	Y
	**OFT**	Total distance traveled (locomotion)	N	N
		% time in center	Y	N
	**MBT**	Number of marbles buried	Y	Y
**Depressive-like** **behavior**	**FST**	Time immobile	Y	Y
**Memory**	**NORT**	STM DR	Y	N
		LTM DR	Y	Y

*Note.* Y: Significant difference/effect found. N: No significant difference/effect found. EPM: Elevated Plus Maze: OFT: Open Field Test. NORT: Novel Object Recognition Test. STM DR: Short-Term Memory Discrimination Ratio. LTM DR: Long-Term Memory Discrimination Ratio. MBT: Marble Burying Test. FST: Forced Swim Test.

## Data Availability

The raw data supporting the conclusions of this article will be made available by the authors on request.

## Supplementary Materials

The following supporting information can be downloaded at: https://www.mdpi.com/article/10.3390/nu18132175/s1. Supplementary Table S1: Post-hoc Sidak’s multiple comparisons test for preference index for salty, sweet and total CAF, for female and male offspring rats; Supplementary Table S2: Post-hoc Sidak’s multiple comparisons test for elevated plus maze, open field test, marble burying test and forced swim test; Supplementary Table S3: Post-hoc Sidak’s multiple comparisons test for Novel Object Recognition Test.

## Abbreviations

The following abbreviations are used in this manuscript:

PND	Postnatal days
CAF	Cafeteria diet
SD	Standard diet
PA	Physical activity
SED	Sedentary
CON	Control
UP	Ultraprocessed
PI	Preference index
FE	Female
MA	Male
EPM	Elevated plus maze
OFT	Open field test
MBT	Marble burying test
FST	Forced swim test
NORT	Novel object recognition test
STM	Short-term memory
LTM	Long-term memory
FAM	Familiar
NOV	Novel
BDNF	Brain-derived neurotrophic factor
