# Supplementary material for: The Effect of Perinatal Exposure to Cafeteria Diet and Physical Activity on Diet Preference, Anxiety-like and Depressive-like Behavior, and Memory in Female and Male Offspring Rats
Source: Nutrients. 2026 Jul 4;18(13):2175. doi: 10.3390/nu18132175 (PMC13364095; doi:10.3390/nu18132175)
Supplement: Supplementary file 1 [file nutrients-18-02175-s001.zip › nutrients-4377634-supplementary.pdf]

Supplementary Table S1: Post-hoc Sidak's multiple comparisons test for preference index for salty, sweet and total CAF, for female and male offspring rats

	FEMALE OFFSPRING				MALE OFFSPRING			
	**Mean Difference**	**95.00% CI of difference**	**Summary**	**Adjusted P Value**	**Mean Difference**	**95.00% CI of difference**	**Summary**	**Adjusted P Value**
**Salty CAF**								
Week 1								
SED+SD vs. SED+CAF	0.1272	0.004953 to 0.2494	*	0.0419	0.1699	0.09573 to 0.2440	***	0.0007
SED+SD vs. PA+CHOW	-0.02826	-0.1942 to 0.1377	ns	0.9901	-0.001714	-0.01546 to 0.01203	ns	0.9981
SED+SD vs. PA+CAF	0.1136	0.02959 to 0.1977	*	0.012	0.06476	-0.01376 to 0.1433	ns	0.1103
SED+CAF vs. PA+SD	-0.1554	-0.2071 to -0.1037	***	0.0002	-0.1716	-0.2412 to -0.1019	***	0.0005
SED+CAF vs. PA+CAF	-0.01351	-0.08471 to 0.05769	ns	0.983	-0.1051	-0.1791 to -0.03110	**	0.0094
PA+SD vs. PA+CAF	0.1419	0.04597 to 0.2378	**	0.0076	0.06647	0.001256 to 0.1317	*	0.046
Week 2								
SED+SD vs. SED+CAF	0.05819	0.02108 to 0.09529	**	0.0056	0.005843	-0.1668 to 0.1785	ns	>0.9999
SED+SD vs. PA+CHOW	-0.01773	-0.2562 to 0.2207	ns	>0.9999	-0.0209	-0.09267 to 0.05087	ns	0.888
SED+SD vs. PA+CAF	0.02463	-0.05872 to 0.1080	ns	0.8816	-0.01146	-0.1204 to 0.09751	ns	0.9993
SED+CAF vs. PA+SD	-0.07591	-0.3284 to 0.1766	ns	0.8737	-0.02674	-0.2679 to 0.2145	ns	0.999
SED+CAF vs. PA+CAF	-0.03356	-0.1239 to 0.05674	ns	0.7437	-0.0173	-0.1112 to 0.07658	ns	0.9853
PA+SD vs. PA+CAF	0.04236	-0.2111 to 0.2958	ns	0.991	0.009443	-0.1655 to 0.1844	ns	>0.9999
Week 3								
SED+SD vs. SED+CAF	-0.03316	-0.1249 to 0.05857	ns	0.7642	-0.0273	-0.1378 to 0.08321	ns	0.9426
SED+SD vs. PA+CHOW	-0.08451	-0.3032 to 0.1342	ns	0.7133	-0.02619	-0.1978 to 0.1455	ns	0.9944
SED+SD vs. PA+CAF	0.06317	-0.003299 to 0.1296	ns	0.0623	0.01136	-0.09344 to 0.1162	ns	0.9991
SED+CAF vs. PA+SD	-0.05136	-0.2505 to 0.1478	ns	0.9311	0.001114	-0.09014 to 0.09237	ns	>0.9999
SED+CAF vs. PA+CAF	0.09633	0.02049 to 0.1722	*	0.0164	0.03866	0.02879 to 0.04853	****	<0.0001
PA+SD vs. PA+CAF	0.1477	-0.01564 to 0.3110	ns	0.0766	0.03754	-0.05712 to 0.1322	ns	0.6919
Week 4								
SED+SD vs. SED+CAF	0.005086	-0.2643 to 0.2745	ns	>0.9999	0.0102	-0.1937 to 0.2141	ns	>0.9999
SED+SD vs. PA+CHOW	-0.005129	-0.1539 to 0.1436	ns	>0.9999	0.08283	-0.03811 to 0.2038	ns	0.2118
SED+SD vs. PA+CAF	0.06111	-0.1249 to 0.2472	ns	0.8269	0.07334	-0.05915 to 0.2058	ns	0.3832
SED+CAF vs. PA+SD	-0.01021	-0.1347 to 0.1142	ns	0.9998	0.07263	-0.01189 to 0.1571	ns	0.0941
SED+CAF vs. PA+CAF	0.05603	-0.09780 to 0.2099	ns	0.7587	0.06314	-0.04663 to 0.1729	ns	0.3488
PA+SD vs. PA+CAF	0.06624	-0.02366 to 0.1561	ns	0.1662	-0.009486	-0.06782 to 0.04885	ns	0.9922
Week 5								
SED+SD vs. SED+CAF	-0.003214	-0.2689 to 0.2625	ns	>0.9999	-0.009443	-0.3354 to 0.3165	ns	>0.9999
SED+SD vs. PA+CHOW	-0.03499	-0.3165 to 0.2465	ns	0.9982	0.0519	-0.1618 to 0.2656	ns	0.9466
SED+SD vs. PA+CAF	0.07381	-0.07672 to 0.2244	ns	0.4975	0.06356	-0.07396 to 0.2011	ns	0.554
SED+CAF vs. PA+SD	-0.03177	-0.1899 to 0.1263	ns	0.9776	0.06134	-0.1903 to 0.3130	ns	0.9458
SED+CAF vs. PA+CAF	0.07703	-0.08521 to 0.2393	ns	0.5284	0.073	-0.1733 to 0.3193	ns	0.8802
PA+SD vs. PA+CAF	0.1088	-0.05557 to 0.2732	ns	0.2356	0.01166	-0.1230 to 0.1463	ns	0.9998
Week 6								
SED+SD vs. SED+CAF	-0.02694	-0.2954 to 0.2415	ns	0.9994	-0.02224	-0.2744 to 0.2299	ns	0.9997
SED+SD vs. PA+CHOW	-0.1907	-0.3045 to -0.07680	**	0.004	-0.09171	-0.2381 to 0.05470	ns	0.2773

SED+SD vs. PA+CAF	-0.166	-0.3394 to 0.007415	ns	0.0604	-0.1341	-0.2893 to 0.02100	ns	0.0918
SED+CAF vs. PA+SD	-0.1637	-0.3251 to -0.002370	*	0.0469	-0.06947	-0.2407 to 0.1018	ns	0.6727
SED+CAF vs. PA+CAF	-0.1391	-0.2547 to -0.02348	*	0.0214	-0.1119	-0.2631 to 0.03930	ns	0.1637
PA+SD vs. PA+CAF	0.02466	-0.03569 to 0.08501	ns	0.6666	-0.04241	-0.08434 to -0.0004844	*	0.0475
Week 7								
SED+SD vs. SED+CAF	-0.1519	-0.3387 to 0.03494	ns	0.1165	-0.1161	-0.2373 to 0.005068	ns	0.0601
SED+SD vs. PA+CHOW	-0.237	-0.3580 to -0.1160	**	0.0017	-0.1123	-0.1215 to -0.1032	****	<0.0001
SED+SD vs. PA+CAF	-0.1001	-0.2288 to 0.02863	ns	0.1374	-0.1421	-0.1925 to -0.09164	***	0.0002
SED+CAF vs. PA+SD	-0.08513	-0.1604 to -0.009829	*	0.0287	0.003757	-0.1099 to 0.1174	ns	>0.9999
SED+CAF vs. PA+CAF	0.0518	-0.08365 to 0.1872	ns	0.7215	-0.02596	-0.1812 to 0.1293	ns	0.991
PA+SD vs. PA+CAF	0.1369	0.05386 to 0.2200	**	0.0043	-0.02971	-0.08336 to 0.02393	ns	0.3828
**Sweet CAF**								
Week 1								
SED+SD vs. SED+CAF	-0.01176	-0.1461 to 0.1225	ns	0.9997	-0.003314	-0.1698 to 0.1632	ns	>0.9999
SED+SD vs. PA+CHOW	0.06951	0.04272 to 0.09631	***	0.0004	0.01426	-0.1044 to 0.1329	ns	0.9985
SED+SD vs. PA+CAF	0.08909	0.05606 to 0.1221	***	0.0003	0.03621	-0.09481 to 0.1672	ns	0.9086
SED+CAF vs. PA+SD	0.08127	-0.06447 to 0.2270	ns	0.3766	0.01757	-0.06438 to 0.09952	ns	0.9696
SED+CAF vs. PA+CAF	0.1008	-0.02728 to 0.2290	ns	0.1315	0.03953	-0.03146 to 0.1105	ns	0.3779
PA+SD vs. PA+CAF	0.01957	-0.006843 to 0.04599	ns	0.1631	0.02196	0.001034 to 0.04288	*	0.0404
Week 2								
SED+SD vs. SED+CAF	-0.06329	-0.1706 to 0.04405	ns	0.3273	-0.0545	-0.1452 to 0.03622	ns	0.3114
SED+SD vs. PA+CHOW	-0.01771	-0.03527 to -0.0001599	*	0.048	-0.08317	-0.3569 to 0.1906	ns	0.8687
SED+SD vs. PA+CAF	-0.06476	-0.1653 to 0.03576	ns	0.2556	-0.0421	-0.2081 to 0.1239	ns	0.9358
SED+CAF vs. PA+SD	0.04557	-0.06073 to 0.1519	ns	0.6239	-0.02867	-0.2393 to 0.1819	ns	0.997
SED+CAF vs. PA+CAF	-0.001471	-0.1490 to 0.1461	ns	>0.9999	0.0124	-0.1131 to 0.1379	ns	0.9995
PA+SD vs. PA+CAF	-0.04704	-0.1310 to 0.03690	ns	0.3721	0.04107	-0.1043 to 0.1864	ns	0.9003
Week 3								
SED+SD vs. SED+CAF	0.03104	-0.05409 to 0.1162	ns	0.7578	0.0162	-0.1149 to 0.1473	ns	0.9982
SED+SD vs. PA+CHOW	0.03816	-0.04985 to 0.1262	ns	0.6136	0.05686	-0.01375 to 0.1275	ns	0.1208
SED+SD vs. PA+CAF	0.08229	-0.04287 to 0.2074	ns	0.2405	0.05591	-0.09936 to 0.2112	ns	0.7669
SED+CAF vs. PA+SD	0.007114	-0.06648 to 0.08071	ns	0.9995	0.04066	-0.02122 to 0.1025	ns	0.241
SED+CAF vs. PA+CAF	0.05124	-0.03543 to 0.1379	ns	0.325	0.03971	-0.01942 to 0.09884	ns	0.2252
PA+SD vs. PA+CAF	0.04413	-0.04694 to 0.1352	ns	0.5089	-0.0009429	-0.09748 to 0.09560	ns	>0.9999
Week 4								
SED+SD vs. SED+CAF	-0.0129	-0.1253 to 0.09955	ns	0.9988	-0.006486	-0.04832 to 0.03535	ns	0.9939
SED+SD vs. PA+CHOW	-0.04154	-0.05669 to -0.02640	***	0.0003	0.009371	-0.03987 to 0.05862	ns	0.9828
SED+SD vs. PA+CAF	0.02731	-0.05731 to 0.1119	ns	0.8371	0.0255	0.004320 to 0.04668	*	0.0213
SED+CAF vs. PA+SD	-0.02864	-0.1555 to 0.09826	ns	0.9615	0.01586	-0.008866 to 0.04058	ns	0.259
SED+CAF vs. PA+CAF	0.04021	-0.0009759 to 0.08140	ns	0.0555	0.03199	0.001297 to 0.06267	*	0.0416
PA+SD vs. PA+CAF	0.06886	-0.03078 to 0.1685	ns	0.2058	0.01613	-0.01463 to 0.04688	ns	0.4335
Week 5								
SED+SD vs. SED+CAF	-0.01293	-0.08107 to 0.05521	ns	0.9831	-0.05557	-0.2519 to 0.1408	ns	0.8997
SED+SD vs. PA+CHOW	0.0339	-0.04983 to 0.1176	ns	0.6744	0.0201	-0.1175 to 0.1577	ns	0.9956
SED+SD vs. PA+CAF	0.06567	-0.1742 to 0.3056	ns	0.9121	0.003986	-0.1594 to 0.1674	ns	>0.9999

SED+CAF vs. PA+SD	0.04683	-0.04391 to 0.1376	ns	0.4487	0.07567	-0.1128 to 0.2641	ns	0.6814
SED+CAF vs. PA+CAF	0.0786	-0.1770 to 0.3342	ns	0.8627	0.05956	-0.2123 to 0.3314	ns	0.9664
PA+SD vs. PA+CAF	0.03177	-0.1356 to 0.1992	ns	0.983	-0.01611	-0.1545 to 0.1223	ns	0.9987
Week 6								
SED+SD vs. SED+CAF	-0.003643	-0.05772 to 0.05044	ns	>0.9999	0.001243	-0.03469 to 0.03718	ns	>0.9999
SED+SD vs. PA+CHOW	0.01466	-0.05230 to 0.08161	ns	0.9665	0.02819	0.02454 to 0.03184	****	<0.0001
SED+SD vs. PA+CAF	0.03374	-0.05268 to 0.1202	ns	0.7048	0.04019	-0.02795 to 0.1083	ns	0.327
SED+CAF vs. PA+SD	0.0183	0.0001763 to 0.03642	*	0.0479	0.02694	-0.01162 to 0.06550	ns	0.1986
SED+CAF vs. PA+CAF	0.03739	-0.06338 to 0.1381	ns	0.7449	0.03894	-0.04164 to 0.1195	ns	0.5114
PA+SD vs. PA+CAF	0.01909	-0.08279 to 0.1210	ns	0.9841	0.012	-0.05569 to 0.07969	ns	0.9879
Week 7								
SED+SD vs. SED+CAF	-0.05999	-0.1624 to 0.04245	ns	0.3332	-0.02574	-0.06852 to 0.01704	ns	0.3101
SED+SD vs. PA+CHOW	-0.1175	-0.3225 to 0.08749	ns	0.3519	-0.09707	-0.2756 to 0.08150	ns	0.4
SED+SD vs. PA+CAF	0.1064	-0.1731 to 0.3859	ns	0.7251	-0.09966	-0.1744 to -0.02494	*	0.0129
SED+CAF vs. PA+SD	-0.05753	-0.1902 to 0.07511	ns	0.6133	-0.07133	-0.2695 to 0.1269	ns	0.7673
SED+CAF vs. PA+CAF	0.1664	-0.1132 to 0.4460	ns	0.3194	-0.07391	-0.1832 to 0.03537	ns	0.2204
PA+SD vs. PA+CAF	0.2239	-0.1359 to 0.5837	ns	0.2826	-0.002586	-0.1243 to 0.1192	ns	>0.9999
**Total CAF**								
Week 1								
SED+SD vs. SED+CAF	0.06967	-0.05622 to 0.1956	ns	0.3835	0.1048	0.04405 to 0.1655	**	0.0034
SED+SD vs. PA+CHOW	0.0288	-0.05548 to 0.1131	ns	0.8025	0.02341	0.002054 to 0.04477	*	0.0331
SED+SD vs. PA+CAF	0.1095	0.06552 to 0.1534	***	0.0004	0.06769	0.06309 to 0.07228	****	<0.0001
SED+CAF vs. PA+SD	-0.04087	-0.1151 to 0.03340	ns	0.3887	-0.08134	-0.1368 to -0.02587	**	0.008
SED+CAF vs. PA+CAF	0.03981	-0.05912 to 0.1388	ns	0.6796	-0.03707	-0.1003 to 0.02615	ns	0.332
PA+SD vs. PA+CAF	0.08069	0.03764 to 0.1237	**	0.0022	0.04427	0.01847 to 0.07008	**	0.0035
Week 2								
SED+SD vs. SED+CAF	-0.01077	-0.09279 to 0.07125	ns	0.9975	-0.01806	-0.08348 to 0.04736	ns	0.9091
SED+SD vs. PA+CHOW	-0.01867	-0.1450 to 0.1077	ns	0.9953	-0.04069	-0.2341 to 0.1527	ns	0.9722
SED+SD vs. PA+CAF	-0.02103	-0.08924 to 0.04718	ns	0.8615	-0.01541	-0.06892 to 0.03809	ns	0.8925
SED+CAF vs. PA+SD	-0.0079	-0.1668 to 0.1510	ns	>0.9999	-0.02263	-0.2526 to 0.2073	ns	0.9995
SED+CAF vs. PA+CAF	-0.01026	-0.1339 to 0.1134	ns	0.9998	0.002643	-0.1063 to 0.1115	ns	>0.9999
PA+SD vs. PA+CAF	-0.002357	-0.09741 to 0.09270	ns	>0.9999	0.02527	-0.1225 to 0.1731	ns	0.9899
Week 3								
SED+SD vs. SED+CAF	-0.001057	-0.05824 to 0.05613	ns	>0.9999	-0.005586	-0.1214 to 0.1102	ns	>0.9999
SED+SD vs. PA+CHOW	-0.02316	-0.1766 to 0.1302	ns	0.9947	0.01531	-0.1058 to 0.1364	ns	0.998
SED+SD vs. PA+CAF	0.07279	-0.01341 to 0.1590	ns	0.1007	0.03357	-0.08365 to 0.1508	ns	0.8949
SED+CAF vs. PA+SD	-0.0221	-0.1344 to 0.09021	ns	0.9798	0.0209	-0.003078 to 0.04488	ns	0.0889
SED+CAF vs. PA+CAF	0.07384	0.004306 to 0.1434	*	0.0383	0.03916	0.008364 to 0.06995	*	0.0163
PA+SD vs. PA+CAF	0.09594	0.002072 to 0.1898	*	0.0454	0.01826	0.0002125 to 0.03630	*	0.0475
Week 4								
SED+SD vs. SED+CAF	-0.003929	-0.08377 to 0.07591	ns	>0.9999	0.001871	-0.1191 to 0.1228	ns	>0.9999
SED+SD vs. PA+CHOW	-0.02334	-0.1054 to 0.05868	ns	0.8975	0.04613	-0.03892 to 0.1312	ns	0.4021
SED+SD vs. PA+CAF	0.04421	-0.01831 to 0.1067	ns	0.1909	0.04944	-0.02533 to 0.1242	ns	0.2363
SED+CAF vs. PA+SD	-0.01941	-0.02318 to -0.01565	****	<0.0001	0.04426	0.005018 to 0.08350	*	0.029

SED+CAF vs. PA+CAF	0.04814	-0.01521 to 0.1115	ns	0.1492	0.04757	-0.01831 to 0.1135	ns	0.1781
PA+SD vs. PA+CAF	0.06756	0.003589 to 0.1315	*	0.0392	0.003314	-0.02908 to 0.03571	ns	0.9994
Week 5								
SED+SD vs. SED+CAF	-0.008771	-0.1511 to 0.1336	ns	>0.9999	-0.04697	-0.2751 to 0.1812	ns	0.9749
SED+SD vs. PA+CHOW	-0.01517	-0.2035 to 0.1732	ns	0.9998	0.006743	-0.05912 to 0.07261	ns	0.9994
SED+SD vs. PA+CAF	0.06799	-0.06690 to 0.2029	ns	0.4712	0.0092	-0.007395 to 0.02580	ns	0.3819
SED+CAF vs. PA+SD	-0.0064	-0.1053 to 0.09253	ns	>0.9999	0.05371	-0.1594 to 0.2668	ns	0.9374
SED+CAF vs. PA+CAF	0.07676	0.01169 to 0.1418	*	0.0235	0.05617	-0.1657 to 0.2781	ns	0.9363
PA+SD vs. PA+CAF	0.08316	0.02711 to 0.1392	**	0.0075	0.002457	-0.05531 to 0.06022	ns	>0.9999
Week 6								
SED+SD vs. SED+CAF	-0.009414	-0.1388 to 0.1199	ns	>0.9999	-0.01331	-0.1737 to 0.1470	ns	0.9998
SED+SD vs. PA+CHOW	-0.08101	-0.1132 to -0.04878	***	0.0004	-0.03291	-0.1048 to 0.03901	ns	0.5634
SED+SD vs. PA+CAF	-0.06109	-0.1623 to 0.04016	ns	0.3078	-0.04249	-0.1170 to 0.03205	ns	0.3568
SED+CAF vs. PA+SD	-0.0716	-0.1711 to 0.02792	ns	0.1803	-0.0196	-0.1321 to 0.09290	ns	0.9889
SED+CAF vs. PA+CAF	-0.05167	-0.1217 to 0.01831	ns	0.1651	-0.02917	-0.1564 to 0.09805	ns	0.9586
PA+SD vs. PA+CAF	0.01993	-0.05568 to 0.09554	ns	0.9246	-0.009571	-0.03603 to 0.01689	ns	0.7636
Week 7								
SED+SD vs. SED+CAF	-0.09637	-0.2380 to 0.04531	ns	0.2165	-0.07219	-0.1204 to -0.02401	**	0.0071
SED+SD vs. PA+CHOW	-0.1656	-0.3271 to -0.004115	*	0.0448	-0.09954	-0.2028 to 0.003686	ns	0.0585
SED+SD vs. PA+CAF	0.01476	-0.1454 to 0.1749	ns	0.9997	-0.1157	-0.1514 to -0.07993	****	<0.0001
SED+CAF vs. PA+SD	-0.06923	-0.1246 to -0.01386	*	0.0177	-0.02736	-0.08734 to 0.03263	ns	0.5666
SED+CAF vs. PA+CAF	0.1111	-0.09502 to 0.3173	ns	0.4077	-0.04349	-0.06079 to -0.02618	***	0.0004
PA+SD vs. PA+CAF	0.1804	-0.03478 to 0.3955	ns	0.1036	-0.01613	-0.08591 to 0.05365	ns	0.9571

Note : CI: Confidence Intervals; ns: non-significant; *p<0.05; **p<0.01; *** p<0.001; **** p<0.0001

Supplementary Table S2: Post-hoc Sidak's multiple comparisons test for elevated plus maze, open field test, marble burying test and forced swim test

	FEMALE OFFSPRING				MALE OFFSPRING			
	**Mean Difference**	**95.00% CI of difference**	**Summary**	**Adjusted P Value**	**Mean Difference**	**95.00% CI of difference**	**Summary**	**Adjusted P Value**
**Elevated Plus Maze**								
% entries to open arms								
CON vs. SED+SD	5.814	-13.62 to 25.25	ns	0.9905	1.037	-13.81 to 15.88	ns	0.9996
CON vs. SED+CAF	15.29	-4.146 to 34.72	ns	0.2154	16.21	1.362 to 31.05	*	0.027
CON vs. PA+SD	1.304	-18.13 to 20.74	ns	>0.9999	0.6206	-14.23 to 15.47	ns	>0.9999
CON vs. PA+CAF	0.0283	-19.40 to 19.46	ns	>0.9999	18.19	3.343 to 33.03	*	0.0104
SED+SD vs. SED+CAF	9.472	-9.197 to 28.14	ns	0.7661	15.17	0.3246 to 30.02	*	0.0433
SED+SD vs. PA+SD	-4.51	-23.18 to 14.16	ns	0.9983	-0.4167	-15.26 to 14.43	ns	>0.9999
SED+SD vs. PA+CAF	-5.786	-24.46 to 12.88	ns	0.9877	17.15	2.305 to 32.00	*	0.0173
SED+CAF vs. PA+SD	-13.98	-32.65 to 4.687	ns	0.2696	-15.59	-30.43 to -0.7413	*	0.0359
SED+CAF vs. PA+CAF	-15.26	-33.93 to 3.412	ns	0.1779	1.981	-12.87 to 16.83	ns	0.995
PA+SD vs. PA+CAF	-1.276	-19.94 to 17.39	ns	>0.9999	17.57	2.722 to 32.41	*	0.0141
% time in open arms								
CON vs. SED+SD	-0.4797	-19.72 to 18.77	ns	>0.9999	2.681	-12.36 to 17.72	ns	0.9999
CON vs. SED+CAF	13.34	-5.901 to 32.59	ns	0.3659	-15.16	-30.20 to -0.1177	*	0.0472
CON vs. PA+SD	2.02	-17.22 to 21.27	ns	>0.9999	1.956	-13.09 to 17.00	ns	>0.9999
CON vs. PA+CAF	-9.728	-28.97 to 9.516	ns	0.7697	-13.12	-28.17 to 1.918	ns	0.1241
SED+SD vs. SED+CAF	13.82	-4.667 to 32.31	ns	0.2716	-17.84	-32.88 to -2.799	*	0.0118
SED+SD vs. PA+SD	2.5	-15.99 to 20.99	ns	>0.9999	-0.7254	-15.77 to 14.32	ns	>0.9999
SED+SD vs. PA+CAF	-9.249	-27.74 to 9.241	ns	0.7802	-15.81	-30.85 to -0.7631	*	0.0341
SED+CAF vs. PA+SD	-11.32	-29.81 to 7.167	ns	0.5355	17.12	2.073 to 32.16	*	0.0173
SED+CAF vs. PA+CAF	-23.07	-41.56 to -4.582	**	0.0072	2.036	-13.01 to 17.08	ns	>0.9999
PA+SD vs. PA+CAF	-11.75	-30.24 to 6.741	ns	0.4847	-15.08	-30.12 to -0.03773	*	0.0491
**Open Field Test**								
Total distance travelled								
CON vs. SED+SD	2.077	-2.425 to 6.578	ns	0.8501	-1.557	-6.241 to 3.128	ns	0.9799
CON vs. SED+CAF	-0.2971	-4.799 to 4.204	ns	>0.9999	-2.203	-6.888 to 2.481	ns	0.8367
CON vs. PA+SD	-1.527	-6.028 to 2.975	ns	0.9765	-0.2786	-4.963 to 4.406	ns	>0.9999
CON vs. PA+CAF	-1.903	-6.404 to 2.599	ns	0.906	-1.969	-6.653 to 2.716	ns	0.9101
SED+SD vs. SED+CAF	-2.374	-6.699 to 1.951	ns	0.6781	-0.6467	-5.331 to 4.038	ns	>0.9999
SED+SD vs. PA+SD	-3.604	-7.929 to 0.7212	ns	0.1605	1.278	-3.407 to 5.963	ns	0.9954
SED+SD vs. PA+CAF	-3.98	-8.305 to 0.3452	ns	0.089	-0.4121	-5.097 to 4.273	ns	>0.9999
SED+CAF vs. PA+SD	-1.23	-5.555 to 3.095	ns	0.9936	1.925	-2.760 to 6.609	ns	0.921
SED+CAF vs. PA+CAF	-1.606	-5.931 to 2.719	ns	0.957	0.2346	-4.450 to 4.919	ns	>0.9999
PA+SD vs. PA+CAF	-0.376	-4.701 to 3.949	ns	>0.9999	-1.69	-6.375 to 2.995	ns	0.9648
% time in center zone								
CON vs. SED+SD	-5.382	-16.58 to 5.819	ns	0.6346	-0.3904	-10.54 to 9.758	ns	>0.9999
CON vs. SED+CAF	6.636	-4.565 to 17.84	ns	0.4368	5.207	-4.941 to 15.36	ns	0.7562

CON vs. PA+SD	4.169	-7.033 to 15.37	ns	0.8143	3.335	-6.813 to 13.48	ns	0.9814
CON vs. PA+CAF	0.1001	-11.10 to 11.30	ns	>0.9999	0.9753	-9.173 to 11.12	ns	>0.9999
SED+SD vs. SED+CAF	12.02	1.256 to 22.78	*	0.0228	5.598	-4.551 to 15.75	ns	0.6742
SED+SD vs. PA+SD	9.551	-1.211 to 20.31	ns	0.1008	3.725	-6.423 to 13.87	ns	0.9606
SED+SD vs. PA+CAF	5.482	-5.280 to 16.24	ns	0.5826	1.366	-8.783 to 11.51	ns	>0.9999
SED+CAF vs. PA+SD	-2.468	-13.23 to 8.294	ns	0.962	-1.872	-12.02 to 8.276	ns	0.9998
SED+CAF vs. PA+CAF	-6.536	-17.30 to 4.226	ns	0.4121	-4.232	-14.38 to 5.917	ns	0.9139
PA+SD vs. PA+CAF	-4.069	-14.83 to 6.693	ns	0.8056	-2.36	-12.51 to 7.789	ns	0.9988
**Marble Burying Test**								
Number of marbles buried								
CON vs. SED+SD	-3.786	-9.749 to 2.178	ns	0.486	-3.571	-9.000 to 1.857	ns	0.4386
CON vs. SED+CAF	-7.071	-13.04 to -1.108	*	0.0119	-8.143	-13.57 to -2.714	***	0.0009
CON vs. PA+SD	0.9286	-5.035 to 6.892	ns	>0.9999	-2.857	-8.286 to 2.572	ns	0.7289
CON vs. PA+CAF	-0.6429	-6.606 to 5.321	ns	>0.9999	-2.714	-8.143 to 2.715	ns	0.7824
SED+SD vs. SED+CAF	-3.286	-9.015 to 2.444	ns	0.6233	-4.571	-10.00 to 0.8574	ns	0.1521
SED+SD vs. PA+SD	4.714	-1.015 to 10.44	ns	0.1717	0.7143	-4.715 to 6.143	ns	>0.9999
SED+SD vs. PA+CAF	3.143	-2.587 to 8.873	ns	0.679	0.8571	-4.572 to 6.286	ns	>0.9999
SED+CAF vs. PA+SD	8	2.270 to 13.73	**	0.0021	5.286	-0.1431 to 10.71	ns	0.0607
SED+CAF vs. PA+CAF	6.429	0.6989 to 12.16	*	0.0197	5.429	-0.0002252 to 10.86	ns	0.05
PA+SD vs. PA+CAF	-1.571	-7.301 to 4.158	ns	0.9951	0.1429	-5.286 to 5.572	ns	>0.9999
**Forced Swim Test**								
Time Immobile								
CON vs. SED+SD	-37.08	-165.5 to 91.37	ns	0.9928	45.96	-43.41 to 135.3	ns	0.7539
CON vs. SED+CAF	-72.37	-200.8 to 56.08	ns	0.6459	58.26	-31.11 to 147.6	ns	0.4511
CON vs. PA+SD	57.22	-71.24 to 185.7	ns	0.875	115.6	26.21 to 204.9	**	0.0049
CON vs. PA+CAF	60.96	-67.49 to 189.4	ns	0.8278	118.6	29.21 to 207.9	**	0.0037
SED+SD vs. SED+CAF	-35.29	-158.7 to 88.13	ns	0.9933	12.3	-77.06 to 101.7	ns	>0.9999
SED+SD vs. PA+SD	94.3	-29.11 to 217.7	ns	0.2467	69.61	-19.75 to 159.0	ns	0.2266
SED+SD vs. PA+CAF	98.04	-25.37 to 221.5	ns	0.2053	72.61	-16.75 to 162.0	ns	0.184
SED+CAF vs. PA+SD	129.6	6.172 to 253.0	*	0.0343	57.31	-32.05 to 146.7	ns	0.4737
SED+CAF vs. PA+CAF	133.3	9.915 to 256.7	*	0.0272	60.31	-29.05 to 149.7	ns	0.4036
PA+SD vs. PA+CAF	3.743	-119.7 to 127.2	ns	>0.9999	3	-86.36 to 92.36	ns	>0.9999

Note : CI: Confidence Intervals; ns: non-significant; *p<0.05; **p<0.01; *** p<0.001

Supplementary Table S3: Post-hoc Sidak's multiple comparisons test for Novel Object Recognition Test

	FEMALE OFFSPRING				MALE OFFSPRING			
	**Predicted (LS) mean difference**	**95.00% CI of difference**	**Summary**	**Adjusted P Value**	**Predicted (LS) mean difference**	**95.00% CI of difference**	**Summary**	**Adjusted P Value**
**Short term memory**								
Familiar - Novel								
CON	-0.2025	-0.3378 to -0.06724	***	0.001	-0.1692	-0.3531 to 0.01483	ns	0.0854
SED+SD	-0.146	-0.2712 to -0.02072	*	0.0151	-0.1112	-0.2815 to 0.05918	ns	0.3705
SED+CAF	0.06243	-0.06283 to 0.1877	ns	0.6532	0.09828	-0.07206 to 0.2686	ns	0.5042
PA+SD	-0.1332	-0.2585 to -0.007957	*	0.032	-0.1457	-0.3161 to 0.02461	ns	0.1271
PA+CAF	-0.006867	-0.1321 to 0.1184	ns	>0.9999	0.08441	-0.08593 to 0.2548	ns	0.6585
**Long term memory**								
Familiar - Novel								
CON	-0.3326	-0.5052 to -0.1600	****	<0.0001	-0.4038	-0.5852 to -0.2224	****	<0.0001
SED+SD	-0.2786	-0.4384 to -0.1188	***	0.0001	-0.4356	-0.6169 to -0.2542	****	<0.0001
SED+CAF	0.3031	0.1433 to 0.4629	****	<0.0001	0.4286	0.2472 to 0.6100	****	<0.0001
PA+SD	-0.196	-0.3558 to -0.03622	**	0.0094	-0.2576	-0.4390 to -0.07627	**	0.0019
PA+CAF	0.4413	0.2815 to 0.6011	****	<0.0001	0.3508	0.1695 to 0.5322	****	<0.0001

Note : CI: Confidence Intervals; ns: non-significant; *p<0.05; **p<0.01; *** p<0.001; **** p<0.0001
